# Control of Microstructure, Trap Levels, and Trap Distribution in HfO_2_ Films Grown by Atomic Layer Deposition

**DOI:** 10.3390/nano16080451

**Published:** 2026-04-09

**Authors:** Seyedeh Mahsa Sharafi, Marco Flores, Himasha Appuhami, Farida A. Selim

**Affiliations:** 1 Chemical Engineering, School for Engineering of Matter, Transport and Energy, Arizona State University, Tempe, AZ 85287, USA; ssharafi@asu.edu; 2 School of Molecular Sciences, Arizona State University, Tempe, AZ 85287, USA; mafloresr@asu.edu; 3 Materials Science and Engineering, School for Engineering of Matter, Transport, and Energy, Arizona State University, Tempe, AZ 85287, USA; maththin@asu.edu

**Keywords:** defects, transition levels, XRD, trap measurements, EPR measurements

## Abstract

HfO_2_ films have become a critical component for advanced electronics and a wide range of applications. However, their implementation requires control of their microstructure and defects, which often act as charge carrier traps, leading to leakage current in devices and hindering their dielectric properties. Here, we deposit HfO_2_ thin films by atomic layer deposition (ALD) on sapphire, Ga_2_O_3_, and InGaO_3_ substrates at low temperature and investigate the dependence of their crystal structure on substrate type, annealing, and thickness. X-ray diffraction measurements showed that alloying Ga_2_O_3_ with a modest amount of Indium transferred HfO_2_ films from amorphous to polycrystalline, an important finding that may be applicable to the deposition of other material systems. The study also presents an interesting approach to measuring shallow and deep traps formed in the films and shows how to control their levels and distributions in the band gap. The measurements reveal that the difference in band gap between the substrate and film, as well as the presence of impurities, strongly influences trap densities and depths. Electron paramagnetic resonance (EPR) measurements were performed to probe the electronic structure of specific point defects detectable by EPR and to correlate these results with trap measurements.

## 1. Introduction

Hafnium dioxide (HfO_2_) has attracted more attention in recent years due to its outstanding physical, chemical, and electrical properties. It features a high dielectric constant (approximately 20), a wide band gap (typically between 5.5 and 6 eV), and a high refractive index ranging from 1.8 to 2.2. Additionally, HfO_2_ is a highly refractory material with excellent thermal stability and mechanical properties [1,2]. These characteristics, along with its good thermodynamic, chemical stability, and broad optical transparency from the ultraviolet (UV) to infrared (IR) regions, make HfO_2_ highly valuable in advanced electronic and optical applications [3,4,5]. On the other hand, HfO_2_ exhibits notable structural properties, which have driven its investigation as a potential ferroelectric material. In particular, it crystallizes in monoclinic, orthorhombic, and tetragonal phases, and, its orthorhombic structure induces a remnant polarization that leads to ferroelectric behavior [6,7]. Given these properties, HfO_2_ thin films have shown potential for use in various applications such as memory devices, sensors, and energy storage systems. For instance, HfO_2_-based memory technologies have demonstrated excellent performance, including high speed, low power consumption, and strong reliability [8,9]. HfO_2_ thin films are increasingly important in both planar and 3D device architectures, serving as gate dielectrics in modern MOSFETs and as insulating layers in memory technologies. Their high laser damage threshold and low optical absorption make them ideal for multilayer optical coatings and protective layers in UV-IR applications [10,11]. For optical applications, particularly in the ultraviolet range, it is essential to use uniform, smooth, dense, and stoichiometric films. In such transparent materials, intrinsic optical losses mainly arise from reflection, while extrinsic losses can result from factors like surface roughness, impurities, non-uniform composition, and structural inconsistencies. Thus, it is important to produce high-purity, high-quality, stoichiometric films and maintain precise control over their composition under different conditions.

The deposition technique strongly influences the structural and optical properties of HfO_2_ thin films. There are different deposition methods, such as sol–gel processing, RF magnetron sputtering, metal–organic chemical vapor deposition (MOCVD), plasma-enhanced chemical vapor deposition (PECVD), physical vapor deposition (PVD), atomic layer deposition (ALD), and chemical vapor deposition (CVD). Among them, atomic layer deposition (ALD) is a widely used method for the deposition of high-quality HfO_2_ thin films due to its excellent conformity and precise thickness control, even on substrates with complex geometries. A key advantage of ALD is its self-limiting surface chemisorption mechanism. The layer-by-layer growth in a self-limiting manner enables ALD to deposit thin films with precise thickness control. This feature ensures that each cycle deposits a consistent amount of material, resulting in excellent repeatability and uniform coverage over large areas. However, the film quality, the resulting structure, and the presence of defects depend on factors such as the deposition temperature and the nature of the substrates [12,13]. Especially, ALD is a highly surface-sensitive deposition method that is impacted by the choice of substrate, as the identity of the substrate influences the amount of precursor that chemisorbs, the driving force for aggregation, and the surface diffusivity [14]. It is important to understand point defects in HfO_2_ films, as the electrical and optical properties of materials are greatly affected by the energy levels of these states in the band gap. Point defects in HfO_2_, especially vacancies and complex defects, hinder its successful development in many applications and degrade the performance of its current devices. Vacancies create deep and shallow trap states that enable trap-assisted tunneling and stress-induced leakage current, accelerating time-dependent dielectric breakdown in high-k materials [15,16,17]. Additionally, at the HfO_2_/semiconductor interface, a high density of interface states scatters carriers, degrades mobility and subthreshold swing, and induces threshold-voltage drift, underscoring the need for careful interface control and passivation [18,19]. Therefore, characterizing and measuring the energy levels of these interface states and defects in the films is highly important.

Recent studies on ALD-grown HfO_2_ thin films have primarily focused on the effects of deposition temperature, thickness, and post-deposition annealing on their structural and electrical properties. However, the role of the substrate in altering crystallinity, defect formation, and trap distributions in HfO_2_ films remains poorly understood. This is especially important for emerging oxide and wide-band gap semiconductors, where interface quality and defect states critically determine device performance. Moreover, although defects in HfO_2_ are known to strongly impact leakage current, reliability, and optical losses, direct experimental characterization of both shallow and deep trap levels and their correlation with microstructure and interface properties remains limited.

In this work, we study atomic layer deposition (ALD) of hafnium oxide (HfO_2_) thin films at a low temperature of 180 °C onto different substrates (sapphire, Ga_2_O_3_, InGaO_3_). The structural and optical properties of the HfO_2_ films are analyzed using X-ray diffraction and UV–Vis spectroscopy. Additionally, defects that influence film characteristics and significantly impact the material’s performance in practical applications are thoroughly investigated. The effect of thickness, post-deposition annealing, and substrate on the crystal structure of the films is studied. Defects and their associated transition levels in the band gap of HfO_2_ films were measured using cryogenic thermally stimulated photoemission spectroscopy (C-TSPS), a recently developed technique by one of the co-authors [20,21]. It is an advanced in-house apparatus of our previous spectrometers, developed to measure transition levels in semiconductors [22,23] and dielectrics [24,25]. Electron paramagnetic resonance (EPR) spectroscopy was employed to investigate paramagnetic defects—i.e., defects containing unpaired electrons—in the HfO_2_ films and their interfaces. Their results helped guide the interpretation of the trap measurements and provided insight into the origin of specific defects acting as charge carrier traps.

By combining studies on substrate effects with advanced defect characterization techniques, this study establishes a direct correlation between substrate properties, film microstructure, and trap distributions. In particular, we show that substrate composition, including Indium alloying, plays a key role in controlling the crystallinity and defect formation of HfO_2_ films. These findings provide new insights into defect engineering and interface control in high-k dielectric materials.

## 2. Materials and Methods

The Cambridge NanoTech Savannah 100 Atomic Layer Deposition System at ASU NanoFab was used for HfO_2_ deposition. Tetrakis(dimethylamido)hafnium (IV) (TDMA-Hf) was used as the precursor for Hf, which can react with water vapor on the substrate surface and form one layer of Hf oxide. The process consists of alternating 15 milliseconds of flashing time from TDMA-Hf and the water source, followed by 15 s of pumping and nitrogen gas purging. In this study, the substrate temperature of HfO_2_ films was 180 °C, and the deposition period was 10, 100, and 500 cycles. Before loading the substrates into the reactor, they were degreased using an ultrasonic bath with ethanol, isopropanol, and deionized water, and then dried with N_2_. It is worth mentioning that sapphire substrates were annealed at high temperature before deposition. In order to obtain information about the effect of substrates on the film growth and properties, the films were simultaneously grown on different substrates, including commercial sapphire, Ga_2_O_3_, and InGaO_3_ (20% In) films previously grown by MOCVD [26,27]. The growth occurs efficiently at relatively low deposition temperatures, and in our work, it was 180 °C, allowing the use of highly reactive precursors while minimizing thermal stress between the deposited films and substrates. By flashing and keeping the precursors separate throughout the coating process, atomic layer control of film growth can be obtained as fine as ~1 Å per cycle (one monolayer per cycle).

The ALD reaction mechanism follows a ligand-exchange pathway, in which the dimethylamido ligands of TDMA-Hf are replaced by surface hydroxyl groups and subsequently hydrolyzed by water to form Hf–O bonds. The chemical reaction is summarized as follows [3]:

(1) Hf[(CH_3_)_2_N]_4_ + 2H_2_O ⟹ HfO_2_ + 4HN(CH_3_)_2_

After deposition, thermal annealing treatments were performed on some films at 600 °C in air for 1 h.

**Figure 1:**
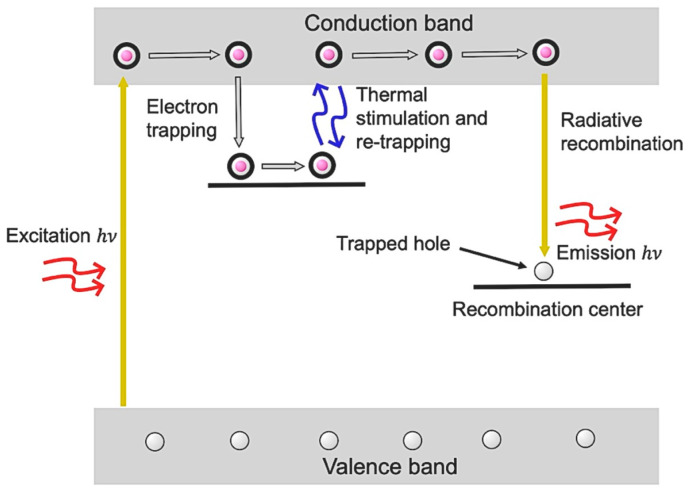
A schematic diagram illustrating the C-TSPS process.

A Woollam M2000 Ellipsometer was used to measure the thickness of thin films on silicon substrates. Optical absorption/transmission spectra were recorded from 190 nm to 800 nm at room temperature using a PerkinElmer ultraviolet-visible-near-infrared (UV Vis-NIR) spectrometer [28]. High-resolution X-ray diffraction (HRXRD) ω/2θ measurements using a Rigaku SmartLab X-ray diffractometer with a Ge 4× (220) monochromator and a Cu Κα X-ray source were performed to study the crystalline structure and phase composition of the films before and after annealing. C-TSPS measurements were carried out using a custom-built spectrometer system, described in detail elsewhere [20]. The detection mechanism is illustrated in the schematic in Figure 1.

The samples were initially cooled to 9 K using a closed-cycle helium cryostat system, a rotary vane vacuum pump, and a temperature controller. Temperature was precisely monitored using a sensor placed beneath the sample stage. During measurements, the samples were placed in a dark, vacuum-sealed chamber and irradiated at 9 K using a broadband photoexcitation source for 15 min. Following excitation, the samples were heated at a constant linear rate (typically 60 °C/min) using an integrated electric heater. The emitted photons were detected by a photomultiplier tube and counted using a photon counting system, generating an intensity versus temperature spectrum commonly referred to as a glow curve [21]. These glow curves often consist of multiple overlapping peaks, each corresponding to distinct trap energy levels within the band gap. By applying a suitable deconvolution method, these individual peaks can be resolved, allowing for the study of key parameters such as activation energy and kinetic orders. In this study, the glow curves were deconvoluted using an R-based computational code developed by Peng et al. [29], which allows for separating overlapping peaks within a complex glow curve.

Continuous wave (CW) EPR spectra were recorded at 293 K using a Bruker ELEXSYS E580 CW X-band spectrometer (Bruker, Rheinstetten, Germany) equipped with a cylindrical mode resonator (ER 4103TM). The magnetic field modulation frequency was 100 kHz with a field modulation amplitude of 1 mT peak-to-peak. The microwave power was 4 mW, the microwave frequency was 9.62 GHz and the sweep time was 168 s. The EPR spectrum was interpreted using a S = 1/2 spin Hamiltonian, [30] H, containing the electron Zeeman interaction with the applied magnetic field B_0_:

(2) $$H=\beta_{e}\;S\cdot g\cdot B_{0}$$

where S is the electron spin operator, *g* is the electronic *g*-tensor, and *β*_e_ is the electron magneton. The EPR spectrum was simulated using EasySpin (version 5.2.36), a computational package developed by Stoll and Schweiger [31] and based on MATLAB (The MathWorks, Natick, MA, USA). The fitting parameters were the *g*-values (*g_x_*, *g_y_*, and *g_z_*) and the line widths (Δ*B*_x_, Δ*B*_y_, and Δ*B*_z_). The fitting procedure was similar to the one previously described by Flores and co-workers [32].

## 3. Results

### 3.1. Film Thickness and Optical Band Gap Measurements

**Figure 2:**
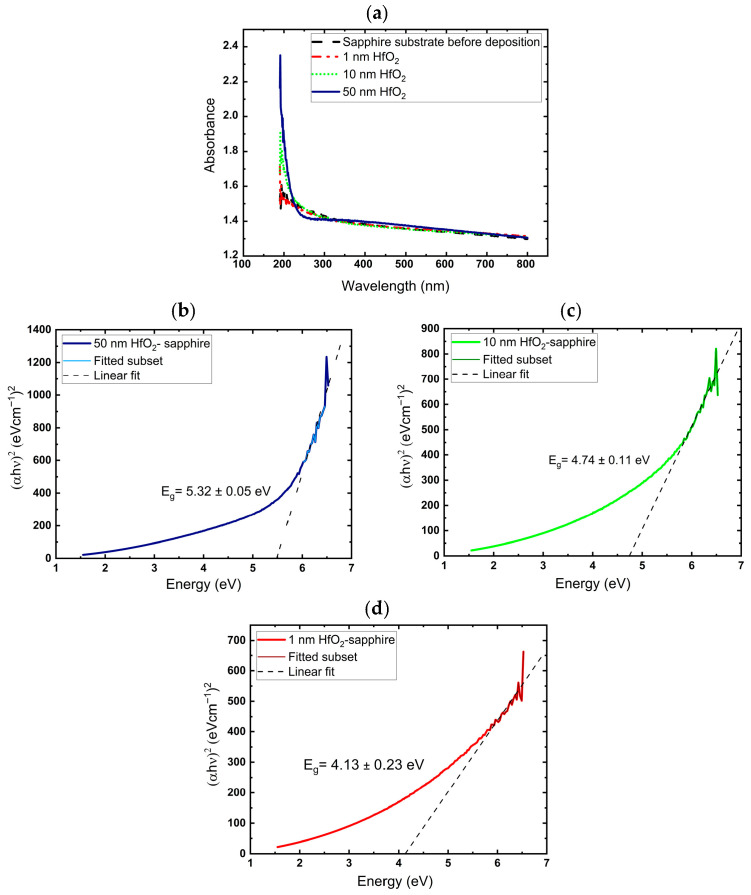
(**a**) Optical absorption spectra of HfO_2_ films with different thicknesses on sapphire substrates. Band gap spectra of (**b**) 50 nm, (**c**) 10 nm, (**d**) 1 nm HfO_2_ film on sapphire substrate.

The thicknesses of the resulting HfO_2_ films on silicon were measured by ellipsometry. For 10, 100, and 500 cycles, thicknesses were 0.98 nm, 10.2 nm, and 50.4 nm, respectively, confirming an ALD rate of 0.1 nm/cycle. The optical absorption measurements of films grown on sapphire substrates with different thicknesses are depicted in Figure 2a.

The band gap energy was determined through the use of a Tauc analysis [33,34], by which the band gap energy is related to the absorption coefficient through the following equation:

(3) $${\left(\alpha h\nu \right)}^{1/\gamma }=A(h\nu -E_{g})$$

where *h* is the Planck constant, *ν* is the photon’s frequency, $E_{g}$ is the band gap energy, and *A* is a constant. The *γ* factor depends on the nature of the electron transition and is equal to 1/2 or 2 for the direct and indirect transition band gaps, respectively. *α* is the absorption coefficient, which depends on the film thickness (length of the absorption media) and absorbance, as given in the following equation:

(4) α = 2.303 *A*/t

Figure 2b–d show the plots of (*αhv*)^2^ versus hν*. E_g_* is determined by extrapolating the straight-line portion of the spectrum to *αhv* = 0. As shown in Figure 2b, the value of the optical energy gap of the 50 nm HfO_2_ thin film is equal to 5.3 eV for the direct transition between valence and conduction bands, which is in good agreement with the previously reported value [4,35].

The measured optical energy gap of the 10 and 1 nm HfO_2_ thin films on sapphire substrates is shown in Figure 2c and Figure 2d, respectively. Although the quantum confinement effect in ultra-thin films can broaden the band gap [36,37], we observed an opposite effect here. This counterintuitive trend can be attributed to defect-related and structural effects in thin films rather than quantum size effects. Insufficient thickness may lead to oxygen deficiency and other defects that introduce mid-gap states, collapsing the apparent band gap. In fact, relatively low thickness can lead to the non-stoichiometry of the film and a drastic reduction in the band gap. Hildebrandt et al. [38] observed a similar narrowing of the band gap of oxygen-deficient HfO_2−x_, of more than 1 eV from 5.7 eV in the stoichiometric case to values as low as 4.5 eV. They reported that this band gap reduction results from oxygen vacancies in HfO_2−x_ or from hafnium vacancies/oxygen interstitials in HfO_2+x_. This band gap narrowing indicates the evolution of gap defect bands, which reduce the total band gap by hybridization with the conduction or valence band [39]. Also, the relatively large error bar for the 1 nm HfO_2_ film mainly results from the known limitations of Tauc analysis for ultra-thin films. At such a thin thickness, the absorption signal is weak, and the extracted absorption coefficient is highly sensitive to uncertainties in thickness, substrate contributions, and the selection of the linear fitting region in the Tauc plot. In addition, for ultra-thin films, interference and thickness–optical constant correlation can introduce additional uncertainty in the band gap estimation. Therefore, the optical band gap obtained for the 1 nm film should be considered as an approximate value with larger uncertainty than for the thicker films.

**Figure 3:**
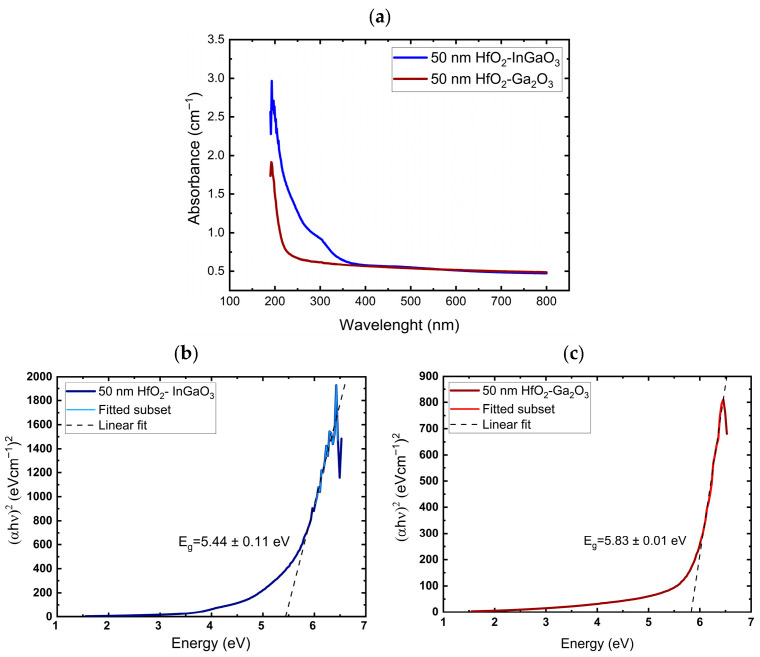
(**a**) Optical absorption and band gap spectra of 50 nm HfO_2_ films on (**b**) InGaO_3_ and (**c**) Ga_2_O_3_ substrates.

The optical absorption spectra of the HfO_2_ thin films (50 nm) on InGaO_3_ and Ga_2_O_3_ substrates are shown in Figure 3a. As shown in Figure 3b,c, the optical band gap of the HfO_2_ films on InGaO_3_ and Ga_2_O_3_ substrates was determined to be approximately 5.44 and 5.83 eV, respectively, which is consistent with the reported values [40]. The lower band edge of the film grown on InGaO_3_ results from the relatively reduced band gap of InGaO_3_.

### 3.2. Structural Properties of the Deposited Films

**Figure 4:**
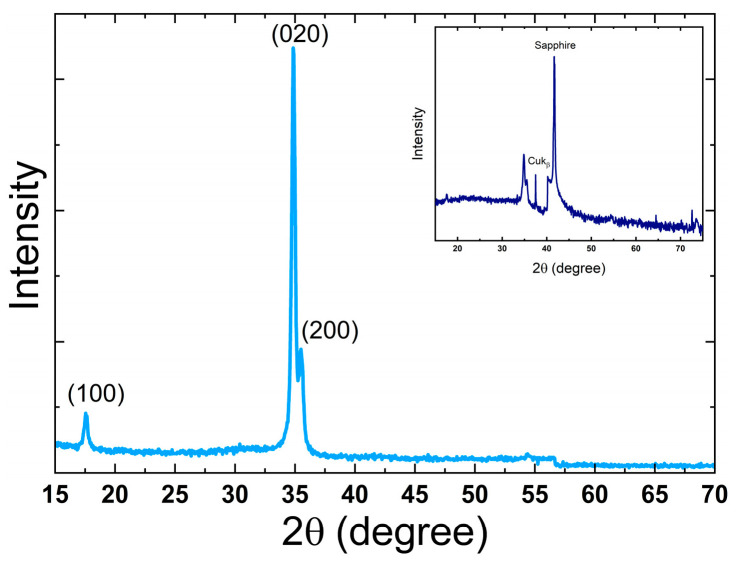
The XRD patterns of the 50 nm HfO_2_ films grown on a sapphire substrate. Major HfO_2_ peaks are indexed. The inset shows the film with an incident angle offset (substrate signal minimized) for clarity.

The HfO_2_ films were grown on sapphire substrates at varying thicknesses (1, 10, and 50 nm) at a temperature of 180 °C. While the 1 and 10 nm thicknesses were amorphous, the thicker film (50 nm) contained crystallites with a monoclinic structure, as shown in Figure 4.

Although many studies consistently report that HfO_2_ films grown by thermal ALD at temperatures below ~200 °C are mostly amorphous on various substrates (Si, glass, metals) [3,13,41,42,43], the deposition in this work at a lower temperature of 180 °C on a sapphire substrate led to crystalline films, which can be attributed to good substrate preparation.

However, the film thickness plays an important role in the crystallinity of ALD HfO_2_. For thin films (< 40 nm), previous works showed that a deposition temperature of 200 °C should result in a mostly amorphous film. The fundamental reason behind that is that a thicker film entails a longer deposition time and more ALD cycles, making nucleation events more probable and resulting in more crystalline films [41]. Hu et al. showed that HfO_2_ films deposited on silicon(100)substrates, even thinner than 130 nm, were predominantly amorphous with a few small crystallites or were polycrystalline with randomly oriented crystalline nuclei [44].

Based on that, we proceeded to investigate only the deposition of 50 nm layers on Ga_2_O_3_ and InGaO_3_ substrates. First, the observed change in the dominant crystallographic orientation from (020) for HfO_2_ films on sapphire to (111) for films on InGaO_3_ can be attributed to substrate-dependent growth mechanisms. The sapphire substrate introduces a relatively large lattice mismatch and interfacial strain, which can favor orientations that minimize strain under kinetically limited growth conditions. In contrast, the InGaO_3_ substrate, particularly due to Indium alloying, modifies the interfacial energy and may reduce the effective lattice mismatch, thereby favoring orientations with lower surface energy. Previous studies have shown that the stability of HfO_2_ phases and orientations depends strongly on epitaxial strain conditions [45]. Also, the (111) orientation is commonly associated with a more thermodynamically stable growth configuration in oxide thin films [46]. Furthermore, differences in nucleation behavior and possible interfacial chemical effects on InGaO_3_ may promote the stabilization of this preferred orientation [47].

**Figure 5:**
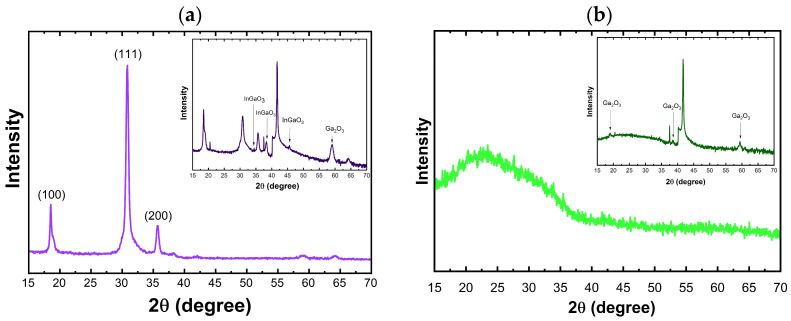
XRD pattern of 50 nm HfO_2_ on (**a**) InGaO_3_, and (**b**) GaO_3_ substrates. The inset shows the film with an incident angle offset (substrate signal minimized) for clarity.

Second, interestingly, as shown in Figure 5a, HfO_2_ deposited films on InGaO_3_ (20% In) showed a polycrystalline structure, whereas they were amorphous on the Ga_2_O_3_ substrate (Figure 5b). Although studies show that the degree of crystallinity of ALD HfO_2_ films depends strongly on film thickness and deposition temperature, with some effect from the substrate [41], our results indicate that the substrate plays a major role in determining the degree of crystallinity.

Ga_2_O_3_, especially in its most common (β) phase, has a monoclinic structure with highly anisotropic lattice parameters (a = 12.21 Å, b = 3.03 Å, c = 5.79 Å) [48], which are quite different from HfO_2_’s monoclinic cell (approximately a ~ 5.12 Å, b ~ 5.17 Å, c ~ 5.29 Å). This results in a significant lattice mismatch, causing interfacial strain and defects that tend to suppress crystalline nucleation and promote amorphous HfO_2_ growth [49]. The formation of crystalline HfO_2_ film on the InGaO_3_ (20% In) substrate is interesting; it may be attributed to the transformation of the single-crystalline monoclinic phase of Ga_2_O_3_ and strain relaxation induced by Indium alloying [50]. Another plausible explanation is interfacial doping, where dopants may stabilize the crystalline phase. If a tiny amount of Indium atoms diffuses from the InGaO_3_ substrate into the first nanometers of HfO_2_, they can reduce the energy barrier for nucleation, which leads to crystalline films. Detecting this is very challenging, but we believe it is a reasonable speculation. Regardless of the underlying mechanism responsible for the observed enhancement in crystallinity, this result is both interesting and potentially novel. It suggests that alloying the substrate, even with a modest composition, can promote crystallinity in the deposited films. This finding may have broader implications, as a similar approach could be applicable to other material systems. To the best of our knowledge, similar results have not been previously reported in the literature.

### 3.3. Thermal Treatment

**Figure 6:**
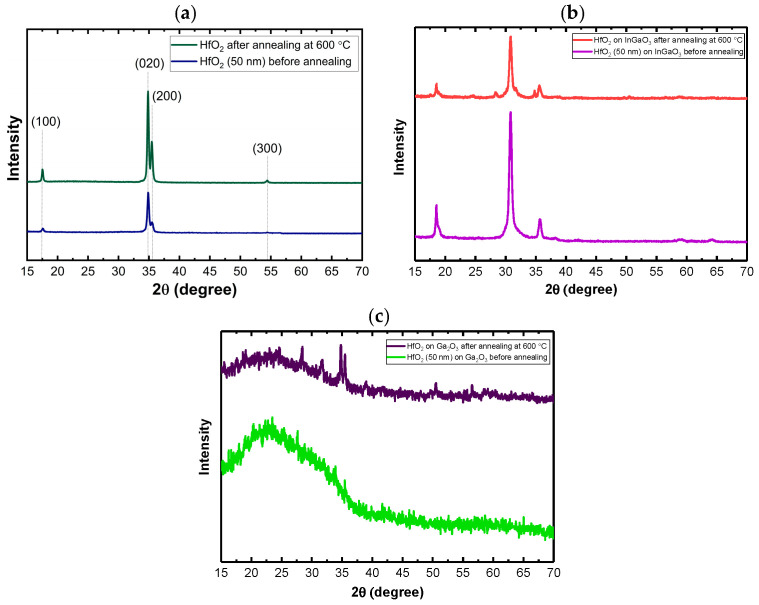
XRD patterns of 50 nm HfO_2_ films on (**a**) sapphire, (**b**) InGaO_3_, and (**c**) Ga_2_O_3_ substrates after annealing at 600 °C. Major HfO_2_ peaks are indexed.

Annealing of the HfO_2_ films converts the as-deposited amorphous material into crystalline phases. Moreover, annealing can be effective in reducing the overall trap densities, and we will investigate this effect in the following section with the C-TSPS measurement. Although the films on the sapphire and InGaO_3_ substrates reported here were crystalline, we performed annealing at 600 °C to investigate possible phase transformations and the effect of thermal treatment on trap densities. Figure 6a shows XRD patterns of HfO_2_ films grown on sapphire substrate annealed at 600 °C. The intensities of the film peaks increase, and their widths decrease with higher annealing temperatures, indicating improved crystallinity and grain growth. However, most of the peaks remain at positions consistent with monoclinic HfO_2_, implying that there is no phase transformation. This behavior is consistent with previous studies, which report that the monoclinic phase is the stable phase for HfO_2_ and typically persists during high-temperature annealing unless specific conditions, such as doping, strain, or a high concentration of defects, are introduced [51,52]. Figure 6b shows XRD patterns of HfO_2_ films grown on InGaO_3_ substrate annealed at 600 °C. After annealing at 600 °C, several new diffraction peaks associated with crystalline monoclinic HfO_2_ appeared. This behavior indicates that annealing improved the crystallinity and led to the formation of additional crystallographic planes. The reduced intensity may also stem from structural relaxation of the film during annealing. Densification or grain growth can reduce the effective scattering, making peaks appear weaker.

As can be seen in Figure 6c and as mentioned earlier, HfO_2_ was completely amorphous on the Ga_2_O_3_ substrate. Annealing led to partial crystallization, as evidenced by the appearance of monoclinic peaks; however, the film remains predominantly amorphous or nanocrystalline, resulting in low-intensity diffraction features.

### 3.4. Trap Measurements

We applied our newly developed method, C-TSPS, to detect shallow and deep trap states in the deposited films. This method is based on performing thermally stimulated photoemission starting in the cryogenic regime and enables measuring both deep and shallow traps with high sensitivity. Currently, no other existing methods offer this capability. The closest comparison—deep-level transient spectroscopy—is limited to traps deeper than 1 eV and requires a p-n junction. In contrast, our method has no such limitations and is applicable to any system without needing a p-n junction. Because of these unique capabilities, our results cannot be directly compared to measurements from standard techniques such as deep-level transient spectroscopy. Moreover, a P-n junction cannot be made on these HfO_2_ films. Because C-TSPS operates in the cryogenic regime, it enables the detection of ultra-shallow traps. Additionally, the use of sensitive photomultiplier tubes (PMTs) and single-photon counting allows efficient measurement of low-density traps in the band gap. Analysis of the temperature-dependent emission or glow curve (C-TSPS) can provide useful information, such as the energy levels (activation energies) of traps/defects, their densities, capture cross sections, and the degree of kinetics, which depends on trapping/detrapping processes [20,21]. A limitation of this technique is its inability to distinguish between donors and acceptors. However, combining other measurements allows us to easily identify the shallow traps as donors or acceptors.

**Figure 7:**
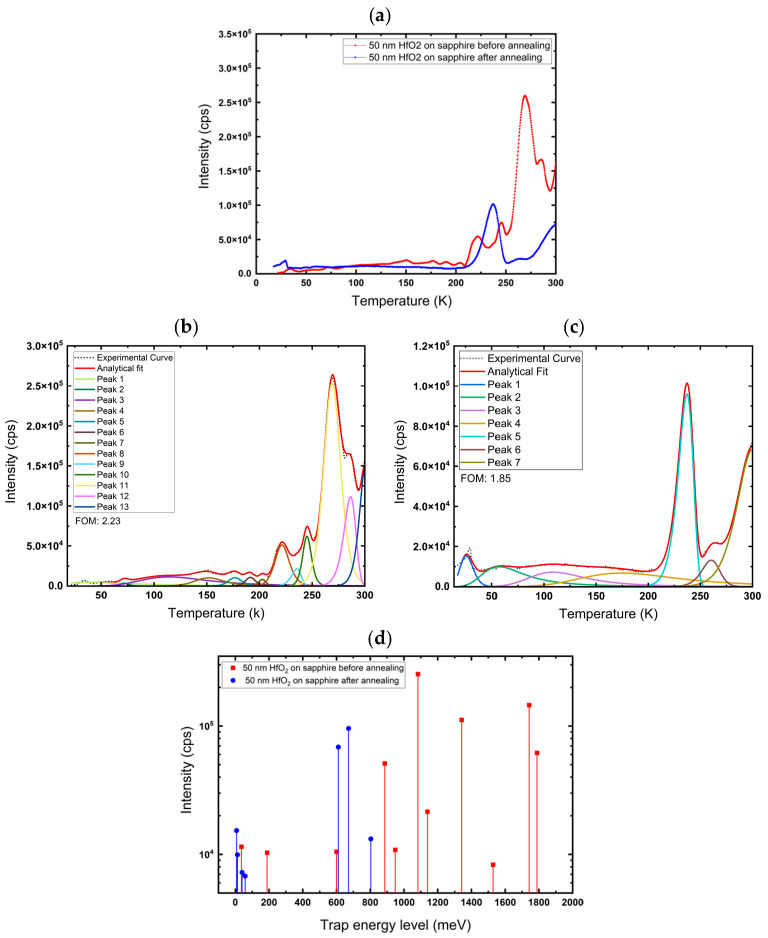
(**a**) Glow curve. (**b**,**c**) Deconvoluted glow curves for deposited 50 nm HfO_2_ on sapphire before and after annealing at 600 °C, respectively. (**d**) Trap energy level of 50 nm HfO_2_ films on sapphire before and after annealing.

**Figure 8:**
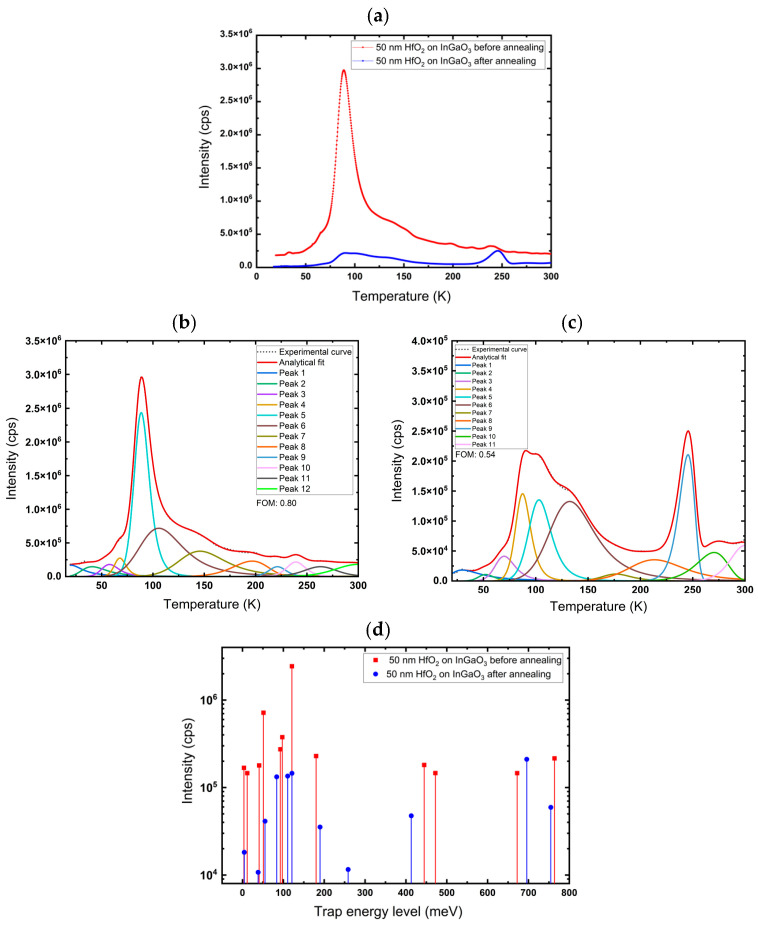
(**a**) Glow curve. (**b**,**c**) Deconvoluted glow curves for deposited 50 nm HfO_2_ on InGaO_3_ before and after annealing at 600 °C, respectively. (**d**) Trap energy level of 50 nm HfO_2_ films on InGaO_3_ before and after annealing.

**Figure 9:**
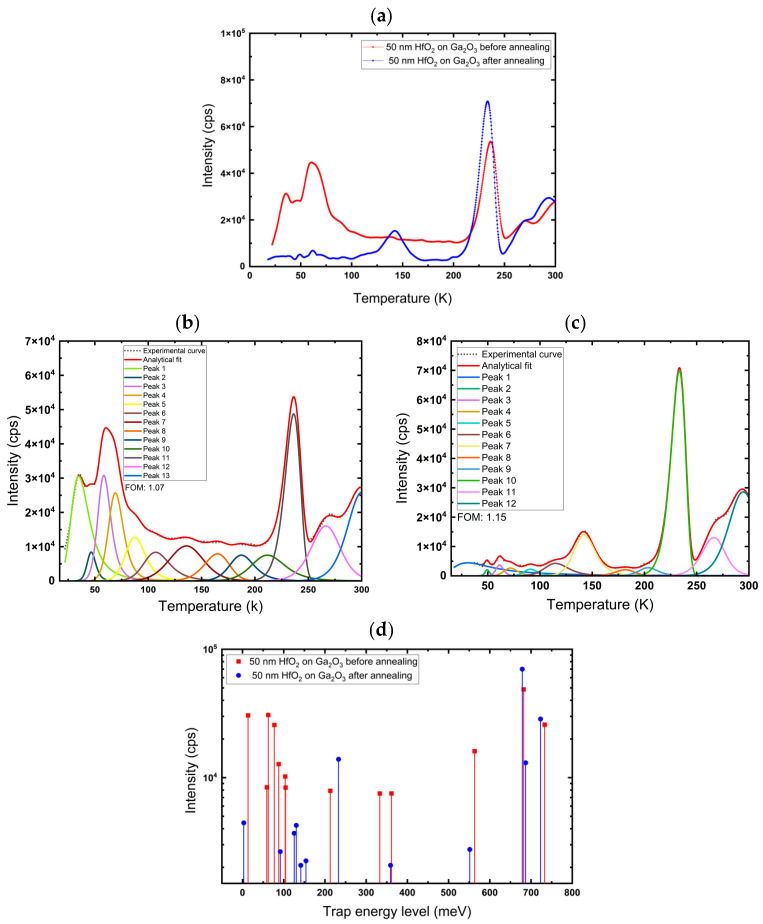
(**a**) Glow curve, (**b**,**c**) Deconvoluted glow curves for deposited 50 nm HfO_2_ on Ga_2_O_3_ before and after annealing at 600 °C, respectively. (**d**) Trap energy level of 50 nm HfO_2_ films on Ga_2_O_3_ before and after annealing for each deconvoluted peak.

The measurements were performed on 50 nm HfO_2_ films, deposited on sapphire, InGaO_3_, and Ga_2_O_3_. The samples were annealed in air at 600 °C for 1 h. After that, C-TSPS spectroscopy was performed again; it was observed (Figure 7, Figure 8 and Figure 9) that the intensity decreased significantly for almost every peak after annealing, which could be explained by a decrease in trap density. This indicates that these traps may be associated with oxygen vacancies (V_O_) related defects and/or disorder-related defects, and annealing in air fills them, thereby reducing their concentration. However, annealing at 600 °C also rearranges the atoms in the lattice, reducing disorder and overall vacancies and interstitials.

Figure 7a depicts the measured glow curves for a 50 nm HfO_2_ film on a sapphire substrate before and after annealing. The procedure for obtaining the glow curves has been described in detail in the Section 2. Figure 7b,c shows the deconvoluted glow curves for the film before and after annealing, plotted as a function of temperature. The fit yielded a Figure of Merit (FOM) of 2.23% before and 1.85% after annealing, indicating a good fit. Thirteen trap levels were identified in the HfO_2_ film on sapphire substrates. After annealing, they were reduced to seven, with lower trap energy levels of 7.56 meV, 13.03 meV, 40.16 meV, 58.36 meV, 671.41 meV, 803.52 meV, and 611.22 meV, and some peaks were completely eliminated (Table 1). However, the glow curve shows a new peak around 240 K with high intensity, which is most likely associated with hafnium vacancy (V_Hf_) or V_Hf_ defect complexes. After annealing, there is a notable reduction in deep traps, and many deep states convert into shallower traps, with much lower activation energies. The energy–intensity mapping in Figure 7d highlights this reduction, showing that the annealed sample has traps with shallower levels. The intensity of each peak corresponds to the number of trapped charge carriers released upon thermal activation.

Figure 8a depicts the glow curve of HfO_2_ on InGaO_3_ before and after annealing at 600 °C. Figure 8b,c shows the deconvoluted glow curves of the films before and after annealing. It reveals a broad distribution of trap levels in both conditions. However, annealing introduces deeper traps, as reflected by higher activation energies (Table 2). These changes suggest that thermal treatment significantly alters the trap intensity, reducing shallow states while activating deeper trap density, possibly complex and interfacial defects. Annealing may enhance diffusion from the InGaO_3_ substrate to the HfO_2_ layer, forming complex defects that may occupy deep states in the band gap (Figure 8d).

Figure 9a depicts the glow curve of HfO_2_ on Ga_2_O_3_ before and after annealing at 600 °C. Figure 9b,c shows the deconvoluted glow curves for the films before and after annealing, plotted as a function of temperature. The fit yielded a Figure of Merit (FOM) of 1.07% before and 1.15% after annealing, indicating a good fit. The glow curve of the HfO_2_ film on the Ga_2_O_3_ substrate shows that the intensity of most peaks decreases after annealing. However, the intensity of the peak around 240 K increases after annealing, which can be attributed to hafnium (Hf) vacancies. This is because annealing in air makes the film richer in oxygen, changing the stoichiometry and leading to the formation of Hf vacancies. Parameters of the deconvoluted peak of 50 nm HfO_2_ on Ga_2_O_3_ substrates before and after annealing at 600 °C are shown in Table 3.

**Figure 10:**
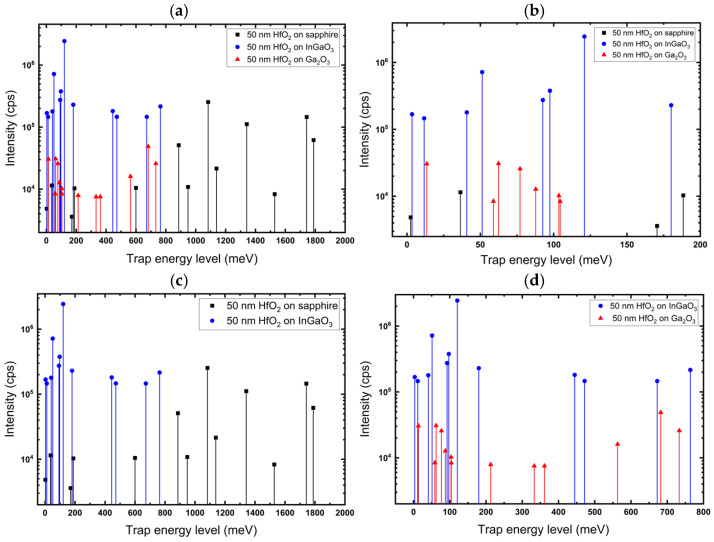
(**a**) Trap energy levels of 50 nm HfO_2_ films on different substrates before annealing, revealing trap depths in the band gap. (**b**) Zoom in the region of zero to 200 meV, showing the several shallow traps formed, especially with Ga_2_O_3_ and InGaO_3_ substrates. (**c**) Comparison of trap energy level of 50 nm HfO_2_ films on InGaO_3_ and sapphire substrates. (**d**) Comparison of trap energy level of 50 nm HfO_2_ films on InGaO_3_ and Ga_2_O_3_ substrates.

To get better insight into the trap levels in HfO_2_ and their interface states, we compare the trap levels and distribution in HfO_2_ films on the three substrates before annealing in Figure 10a,b. Clearly, the trap levels in HfO_2_ on sapphire are much deeper states. This can be explained due to the large band gap difference between sapphire and HfO_2_, and interface states being far from either band edge. These states have been eliminated after air anneal, supporting their association with O-vacancies related defects. The band gap difference between HfO_2_ and Ga_2_O_3_/InGaO_3_ is smaller, resulting in shallower interface states, as shown in Figure 10a. The trap state, around 700 meV in HfO_2_, can be attributed to a Hf vacancy. This conclusion is supported by the increase in its intensity after annealing in air.

In Figure 10c,d, HfO_2_/sapphire and HfO_2_/InGaO_3_ were compared to further understand the effect of the band gap difference between the substrate and film on interface states. The $E_{g}^{sapphire}-E_{g}^{{HfO}_{2}}=4.7$ leading to a deeper interface state, while $E_{g}^{{HfO}_{2}}-E_{g}^{{InGaO}_{3}}=1.1$ leading to a higher concentration of shallower interface states. In Figure 10d, the interface states between HfO_2_/Ga_2_O_3_ and HfO_2_/InGaO_3_ were compared. Since the difference between the band gap of Ga_2_O_3_ and InGaO_3_ (20%In) is modest, we focus here on studying the effect of the crystallinity of HfO_2_ films, as HfO_2_ on Ga_2_O_3_ is amorphous and on InGaO_3_ is polycrystalline. It can be seen that higher concentrations of traps are formed in HfO_2_/InGaO_3_, implying that the amorphous nature of the film did not lead to higher trap densities. We believe that the presence of Indium impurities plays a significant role in leading to the formation of new, deeper complexes involving the Indium impurity.

### 3.5. EPR Measurements

**Figure 11:**
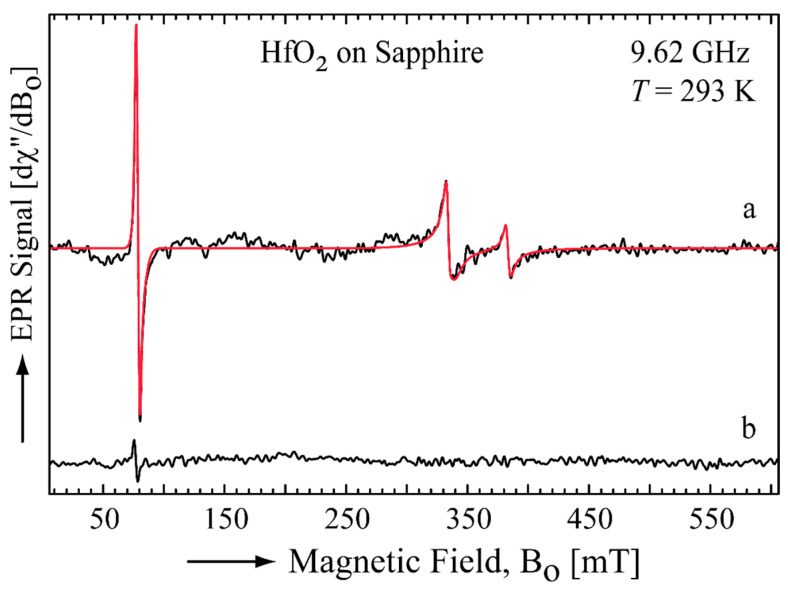
Experimental (black line) and simulated (red line) X-band EPR spectra of a 50 nm HfO_2_ film on sapphire at room temperature (293 K) before annealing (**a**) and after annealing (**b**).

To obtain information on the electronic structure of defects acting as traps, X-band (9.62 GHz) electron paramagnetic resonance (EPR) spectra of HfO_2_ films on sapphire substrates were recorded at room temperature (293 K). Figure 11 shows the EPR spectrum of a 50 nm HfO_2_ film on sapphire (Al_2_O_3_) (black line), which contains one signal around 80 mT (low magnetic field) and two signals within 300 mT and 400 mT. The later ones are consistent with the presence of two S = 1/2 species since they are centered around the magnetic field value corresponding to *g* = 2.0, whereas the signal at low magnetic field corresponds to a S > ½ species.

To obtain the EPR parameters, the respective spin Hamiltonian (see Section 2) was fitted to the data (Figure 11, red line). The EPR signal around 340 mT was well-fit considering a S = ½ species with a rhombic *g*-tensor (*g_x_* = 2.058, *g_y_* = 2.040 and *g_z_* = 1.899) which corresponds to O^2−^ defects. Similar defects have been observed in HfO_2_ films on Si [53]. On the other hand, the signal around 380 mT was fit taking into account a S = ½ species with an axial *g*-tensor (*g*_//_ = 1.898 and *g*_⊥_ = 1.790) which regards to Hf^3+^ occupying a substitutional Al^3+^ site [54]. It is suggested that the substitution occurred at the interface between the film and the substrate. The signal at low magnetic field was assigned to high-spin Fe^3+^ (S = 5/2) and due to trace Fe commonly present in non-ultra-high purity sapphire as the one used in this work. The signals described above disappeared or substantially decreased after thermal annealing.

As depicted in Figure 11, after annealing in air at 600 °C, the O^2−^ and Hf^3+^ signals disappear and the Fe^3+^ signal intensity is significantly reduced. These changes indicate that the as-grown films contain oxygen-related and cation-related defect centers that are removed or transformed during annealing through oxidation and structural relaxation. This behavior well correlates with the substantial reduction in deep trap density observed in our measurements, suggesting that the annealing process passivates defect complexes responsible for deep charge trapping rather than simply filling isolated oxygen vacancies. In particular, the Hf^3+^ centers occupying Al^3+^ sites and the Fe^3+^ impurities in the sapphire substrate are likely associated with interface-related traps that contribute to the deep trap distributions we measure in HfO_2_/sapphire before annealing.

## 4. Conclusions

In this study, HfO_2_ was grown on different substrates (sapphire, Ga_2_O_3_, and InGaO_3_) at a low temperature (180 °C). The substrate type and film thickness strongly influence the crystallinity and optical band gap of the films. X-ray diffraction measurement showed that alloying Ga_2_O_3_ substrate with a small amount of Indium transferred HfO_2_ films from amorphous to polycrystalline. We believe this finding is of great interest in the effect of substrate composition on thin film deposition. Additionally, post-deposition annealing at 600 °C further enhances grain growth and crystallinity of the films, as confirmed by X-ray diffraction, which demonstrates the stability of the monoclinic phase. The C-TSPS measurement, a new approach, provided us with information about traps and how we can control their levels and distributions in the band gap. The study showed that annealing significantly reduces both shallow and deep trap states in the deposited films and revealed how the band gap difference and impurities impact trap density and levels. EPR analysis revealed the presence of O_2_^−^ defects and substitutional Hf^3+^ centers at the HfO_2_/sapphire interface, along with trace Fe^3+^ impurities from the substrate, all of which were eliminated after thermal annealing. Finally, it is important to note that EPR spectroscopy is limited to detecting defects that are paramagnetically active centers. Therefore, the defects identified by EPR represent only a subset of the broader range of defects that may exist in the films and act as charge carrier traps.

Post-deposition annealing in an oxygen-containing environment is highly effective in reducing both shallow and deep trap densities by passivating oxygen-vacancy-related defects and improving structural order. However, annealing conditions must be carefully optimized, as they may also introduce or activate defect complexes depending on the substrate and interfacial diffusion processes. Therefore, achieving high-quality HfO_2_ films with low trap densities requires a combined approach involving appropriate substrate engineering, controlled deposition conditions, and optimized annealing treatments to balance crystallinity and defect passivation for specific device applications.

## Figures and Tables

**Table 1:** Deconvoluted peak parameters of 50 nm HfO_2_ on sapphire substrates before and after annealing at 600 °C. P1 to P13 represent the peak numbers.

	Trap Activation Energy (meV)		Peak Temperature (T_m_) (K)		Kinetic Order (b)	
Peak Numbers	Before Annealing	After Annealing	Before Annealing	After Annealing	Before Annealing	After Annealing
P1	2.50	7.56	36	25	2.00	1.14
P2	170.55	13.03	72	57	2.00	2.00
P3	36.43	40.16	114	108	2.00	2.00
P4	188.38	58.36	152	174	2.00	2.00
P5	599.76	671.41	177	237	2.00	1.00
P6	948.37	803.52	191	260	2.00	1.40
P7	1528.56	611.22	203	300	2.00	1.12
P8	886.27		221		2.00	
P9	1139.10		235		1.15	
P10	1788.86		245		2.00	
P11	1083.15		269		1.86	
P12	1341.80		286		1.14	
P13	1742.31		300		1.00	

**Table 2:** Deconvoluted peak parameters of 50 nm HfO_2_ on InGaO_3_ substrates before and after annealing at 600 °C. P1 to P13 represent the peak numbers.

	Trap Activation Energy (meV)		Peak Temperature (T_m_) (K)		Kinetic Order (b)	
Peak Numbers	Before Annealing	After Annealing	Before Annealing	After Annealing	Before Annealing	After Annealing
P1	3.44	4.42	20	30	2.00	2.00
P2	11.69	38.32	41	52	2.00	2.00
P3	40.76	55.21	58	70	2.00	2.00
P4	92.62	121.13	68	87	2.00	2.00
P5	120.95	110.50	88	103	2.00	2.00
P6	51.25	83.69	106	133	1.93	2.00
P7	97.43	258.67	146	177	2.00	2.00
P8	180.14	189.91	197	213	1.00	2.00
P9	672.32	695.86	221	246	1.81	1.00
P10	763.70	413.10	239	270	2.00	1.00
P11	472.08	754.82	263	300	1.66	2.00
P12	444.64		300		2.00	

**Table 3:** Deconvoluted peak parameters of 50 nm HfO_2_ on Ga_2_O_3_ substrates before and after annealing at 600 °C. P1 to P13 represent the peak numbers.

	Trap Activation Energy (meV)		Peak Temperature (T_m_) (K)		Kinetic Order (b)	
Peak Numbers	Before Annealing	After Annealing	Before Annealing	After Annealing	Before Annealing	After Annealing
P1	13.15	3.05	35	31	2.00	2.00
P2	58.91	141.41	47	49	1.85	2.00
P3	62.43	125.35	58	61	2.00	2.00
P4	77.10	91.61	69	71	2.00	2.00
P5	87.81	154.03	87	90	1.85	2.00
P6	104.44	130.43	107	115	2.00	2.00
P7	103.57	233.06	136	142	1.35	2.00
P8	212.96	359.16	165	182	1.25	1.41
P9	361.31	551.53	187	203	2.00	2.00
P10	332.99	678.76	212	233	2.00	1.02
P11	682.01	686.99	236	266	1.08	1.58
P12	563.03	723.01	266	294	1.63	2.00
P13	733.05		300		1.53	

## Data Availability

The original contributions presented in this study are included in the article. Further inquiries can be directed to the corresponding author.
